# Temporal patterns of functional anti-dengue antibodies in dengue infected individuals with different disease outcome or infection history

**DOI:** 10.1038/s41598-022-21722-2

**Published:** 2022-10-25

**Authors:** Hoa Thi My Vo, Vinit Upasani, Heidi Auerswald, Sokchea Lay, Sotheary Sann, Axelle Vanderlinden, Sreymom Ken, Sopheak Sorn, Sowath Ly, Veasna Duong, Philippe Dussart, Tineke Cantaert

**Affiliations:** 1 Immunology Unit, Institut Pasteur du Cambodge, The Pasteur Network, Phnom Penh, Cambodia; 2 Virology Unit, Institut Pasteur du Cambodge, The Pasteur Network, Phnom Penh, Cambodia; 3 Epidemiology and Public Health Unit, Institut Pasteur du Cambodge, The Pasteur Network, Phnom Penh, Cambodia; 4 Present Address: Centre for Tropical Medicine, Oxford University Clinical Research Unit, Ho Chi Minh, Vietnam; 5 Present Address: The Pasteur Network, Institut Pasteur de Madagascar, Antananarivo, Madagascar

**Keywords:** Viral infection, Humoral immunity

## Abstract

Heterotypic secondary dengue virus (DENV) infection is a risk factor for the development of severe disease. To assess the contribution of the developing polyclonal humoral immune response to the course of acute infection, we have determined anti-DENV IgG titers, neutralizing antibodies, percentages of antibodies binding to DENV-infected cells and antibody‑dependent enhancement (ADE) to the infecting serotype in DENV-infected Cambodian children (*n* = 58), ranging from asymptomatic dengue to severe disease. The results showed that ADE titers are highest against the infecting serotype during heterotypic secondary DENV-2 infection. Moreover, IgG titers, neutralizing antibodies and ADE titers against the infecting serotype peak at D10 and are maintained until D60 after laboratory-confirmed secondary DENV infection. Anti-DENV IgG titers and the magnitude of the functional antibody response were higher in secondary DENV-infected patients compared to primary infected patients. No differences in antibody titers, neutralizing or enhancing antibodies could be observed between asymptomatic or hospitalized patients between 6 and 8 days after laboratory-confirmed DENV-1 infection. However, at this time point, the level of IgG bound to DENV-infected cells was associated with disease severity in hospitalized patients. Taken together, our data offer insights for more comprehensive interpretation of antibody response profile to natural infection and its correlation to disease outcome.

## Introduction

Dengue infections are the most widespread mosquito-borne viral infections in humans^1^. It has been estimated that around 390 million infections occur in the tropical and subtropical regions each year and approximately 96 million infections present clinically^2^. DENV is transmitted by mosquitoes of the *Aedes* species, such as *Aedes aegypti* and *Aedes albopictus*. DENV is a single positive-stranded RNA virus belonging to the *Flavivirus* genus. The group of DENVs consists of four serotypes, DENV-1 to DENV-4, which share 65-80% homology in their genetic sequence^3^. In fact, multiple dengue serotypes co-circulate in hyperendemic regions, which creates complications in the monitoring of dengue epidemiology and challenges the development of a vaccine to prevent infection against all four serotypes^4–6^.

Infections with DENV result in either asymptomatic or inapparent infection, self-limiting dengue fever (DF) or might result in life-threatening severe diseases, dengue hemorrhagic fever (DHF) and dengue shock syndrome (DSS) in about 0.5% of the cases^6^. Primary infection with any DENV serotype induces antibodies with potent protective capacity against homotypic re-infection but also elicits cross-reactive antibodies against other serotypes. While protection against homotypic secondary infection is often long-lasting, cross-protecting antibodies against different serotypes are short-lived, with a half-life from several months to 3 years^7–10^.

Severe dengue, in which hemorrhage, thrombocytopenia, vascular leakage and shock are the major clinical signs and possible cause of death in those patients, occurs almost exclusively in patients infected with a different dengue serotype^11–14^. Moreover, infants born to mothers immune to dengue are likewise more susceptible to severe dengue in the first year after birth^15^. The one commercially available vaccine is recommended by WHO for people who had a confirmed DENV infection prior vaccination, since dengue-naïve individuals who receive the vaccine seem to be at risk of severe diseases and hospitalization when being naturally infected after vaccination^16^.

DENV-specific antibodies can neutralize the virus but also could possibly enhance dengue immunopathogenesis depending on the titer, affinity, avidity and targeted epitopes of the immunoglobulins. Indeed, ADE has been proposed as a mechanism to explain severe dengue disease in seropositive individuals. Here, low affinity antibodies, serotype cross-reactive antibodies or sub-neutralizing concentrations of antibodies acquired during primary infection can increase viral uptake via Fc receptors expressed on target cells such as monocytes, macrophages and dendritic cells. Dengue plasma with neutralizing capacity or neutralizing anti-dengue monoclonal antibodies, when highly diluted, can also have the potential to enhance DV infection in in vitro models^17–20^. Indeed, the ADE hypothesis was observed in studies showing that the presence of low to intermediate titers of pre-existing dengue antibodies correlated with increased risk of development of severe dengue in secondary infected children^18,19^. Specific antibodies targeting the fusion loop of the envelop protein (E) and the other surface protein (prM) have been identified to promote ADE in vitro and in animal models^17,21,22^ and structural determinants of the virus, such as its maturation status, also have an impact on ADE^23,24^.

Next to antibody dependent enhancement, cross-reactive T cells, the infecting serotype and timing between sequential infections can affect the outcome of infection^7,9^. At present, there are no good strategies or predictive markers to identify immune-enhanced dengue disease in general or antibody-dependent enhancement in particular. Antibody titers as well as their affinity and avidity vary greatly from person to person and over time. Therefore, the neutralizing versus enhancing properties of these antibodies also varies. In addition, during acute secondary infection, both pre-existing anti-DENV IgG and newly formed IgG co-circulate. Here, pre-existing IgG antibodies are directed against the previous infecting serotype. Newly formed IgGs are produced by plasmablasts which are mainly derived from memory B cells. In general, these memory B cells are either directly activated, and hence retain binding specificity to the previous infecting serotype, or have re-entered germinal center reactions for further maturation and selection and are hence directed against the current infecting serotype^18,25,26^. It is this polyclonal antibody response that will contribute to either protection through virus neutralization and antibody-dependent effector functions, or enhancement of infection through different ADE mechanisms^27–30^. Since antibodies can exert both protective and potentially detrimental functionalities, these need to be assessed side by side. Therefore, in this study, we aim to understand the kinetics of the anti-DENV antibody titers, neutralizing antibodies, antibodies binding to infected cells and enhancing antibodies during the acute phase of infection and their association to disease severity.

## Methods

### Ethics statement

Ethical approval for the study was obtained from the National Ethics Committee of Health Research of Cambodia. Written informed consent was obtained from all participants or the guardians of participants under 16 years of age prior to inclusion in the study. All experiments were performed in accordance with relevant guidelines and regulations.

### Patient recruitment

Briefly, dengue cases were identified from hospitalized patients presenting with acute dengue-like illness at Kampong Cham Provincial hospital between June and October in 2018 (cohort 1) and at the same hospital and two district hospitals in Kampong Cham province in 2012–2013 (cohort 2). Blood samples were obtained at hospital entry (D0) for DENV detection by RT-qPCR. To identify asymptomatic dengue (ASD) cases, we collected biological specimen from individuals using a household investigation approach around the index cases identified in hospitals in 2018 (cohort 1) and 2012–2013 (cohort 2)^31,32^. Household members of DENV index cases tested positive for DENV by RT-qPCR (D0) but without any clinical symptoms were included. These individuals were classified as ASD at the end of a 10-day follow-up period. In detail, these individuals were questioned about history of symptoms four days before D0 and were followed up until ten days after inclusion for the occurrence of symptoms (including but not limited to fever, rash, headache, retro-orbital pain). For cohort 1, hospitalized patients were included between 1 and 6 days after onset of fever and two follow-up samples were collected at day 10 (D10) and day 60 (D60) after inclusion. ASD cases were sampled at D0, D10 and D60. For cohort 2, hospitalized patients were included at hospital entry, 2–6 days after onset of fever and an additional blood sample was obtained four days later (6–10 days after onset of fever). ASD cases were sampled at D0 and D7. Hospitalized patients were classified according to the WHO 1997 classification scheme in dengue fever (DF), dengue hemorrhagic fever (DHF) or dengue shock syndrome (DSS)^33,34^. Demographics and clinical parameters of patients included in both cohorts are summarized in Tables 1 and 2.

**Table 1:** Demographic and clinical characteristics of cohort 1.

Cohort 1	ASD	DF	Overall
	(*N* = 5)	(*N* = 13)	(*N* = 18)
**Age**			
Mean (SD)	12.2 (1.9)	11.4 (2.1)	11.6 (2.6)
**Gender**			
F	1 (20%)	6 (46%)	7 (39%)
M	4 (80%)	7 (54%)	11 (61%)
**Serotype**			
DENV-2	5 (100%)	13 (100%)	18 (100%)
**Immune status**			
Secondary	5 (100%)	13 (100%)	18 (100%)
**Day of Fever**			
Mean (SD)	NA	2.62 (1.3)	2.62 (1.326)
**Viral load**			
Mean (SD)	1.20E + 08 (2.67E + 08)	1.00E + 08 (3.61E + 08)	1.06E + 08 (3.30E + 08)
**Day of Sampling**			
Mean (SD)	0(0), 10 (0), 60 (0)	0(0), 10 (0), 60 (0)	0(0), 10 (0), 60 (0)
**No of samples**			
D0	5	13	18*
D10	4	11	15*
D60	4	10	14*

NA: not applicable.

*Among those, there are 12 paired-plasma samples across D0,D10 and D60.

**Table 2:** Demographic and clinical characteristics of cohort 2.

Cohort 2	ASD	DF	DHF	DSS	Overall
	(*N* = 8)	(*N* = 13)	(*N* = 13)	(*N* = 6)	(*N* = 40)
**Age**					
Mean (SD)	10.0 (4.1)	8.68 (3.3)	9.23 (3.1)	10.7 (3.5)	9.43 (3)
**Gender**					
Female	3 (38%)	7 (54%)	6 (46%)	2 (33%)	18 (45%)
Male	5 (62%)	6 (46%)	7 (53%)	4 (66%)	22 (55%)
**Serotype**					
DENV-1	8 (100%)	13 (100%)	12 (92.3%)	4 (66.7%)	37 (92.5%)
Undetermined	0 (0%)	0 (0%)	1 (7.7%)	2 (33.3%)	3 (7.5%)
**Immune Status**					
Primary	4 (50.0%)	6 (46.2%)	0 (0%)	0 (0%)	10 (25.0%)
Secondary	4 (50.0%)	7 (53.8%)	13 (100%)	6 (100%)	30 (75.0%)
**Day of Fever**					
Mean (SD)	NA	3.6 (1.3)	4.5 (0.8)	4.3 (0.5)	4.1 (1.1)
**Viral load**					
Mean (SD)	5.99E + 03 (1.06E + 03)	5.75E + 07 (9.1E + 07)	1.18E + 07 (7.27E + 04)	2.28E + 04 (2.73E + 04)	2.47E + 07 (6.13E + 07)
**Day of Sampling**					
Mean (SD)	7.00 (0)	7.00 (0)	7.00 (0)	7.00 (0)	7.00 (0)

NA: not applicable.

### Laboratory diagnosis

Plasma specimens obtained from patients were tested for presence and serotype of DENV using a RT-qPCR^35^ at the Institut Pasteur in Cambodia (IPC), the reference laboratory for arboviral diseases in Cambodia. Only DENV RT-qPCR positive cases were included for further analysis. In 2012–2013, DENV-1 was the most prevalent serotypes circulating in Cambodia, whereas in 2018 DENV-2 was most prevalent. Anti-DENV IgM was measured with an in-house IgM-capture ELISA (MAC-ELISA)^33,36^ and total anti-DENV antibodies using hemagglutination inhibition (HI) assay for DENV, as previously described^37^, to determine primary/secondary DENV infection as per WHO criteria^38^. Primary infections were characterized by the presence or absence of HI antibodies in acute-phase samples (or first samples for inapparent infection) and by low titers with a fourfold rise of HI antibodies (≤ 1:1280) in serum from the convalescence phase (or second samples for inapparent infection) with an elapsed time of at least 7 days between the two collected samples. Conversely, secondary infections were defined by the presence of HI antibodies in acute-phase samples (or first samples for inapparent infection) and by high titers with a fourfold rise of HI antibodies (≥ 1:2560) in serum from the convalescence phase (or second samples for inapparent infection).

### DENV IgG ELISA

DENV-specific IgG antibodies in patient plasma samples were measured with the commercially available Panbio Dengue IgG Indirect ELISA (PanBio) according to the manufacturer’s instructions. The antigen for this ELISA is unknown and proprietary information. Antibody titers were calculated from a dilution range of the provided positive control. Data is reported as Arbitrary Units/ml (AU/ml). In this assay, 12 paired-plasma samples collected from 12 individuals in cohort 1 and 40 plasma samples in cohort 2 were tested.

### Virus production

Dengue virus, DENV-1 (Hawaii strain, GenBank: KM204119), DENV-2 (New Guinea C strain, GenBank: AF038403), DENV-3 (H87 strain, GenBank: M93130) and DENV-4 (H241 strain, Genbank: AY947539) are used as reference strains in this study and produced under BSL2 safety conditions. Briefly, 8×10^6^ *Aedes albopictus* C6/36 cells were seeded in a 75 cm^2^ flask and grown overnight at 28℃, and infected with virus at an MOI of 0.1 and cultured for 5–7 days at 28 °C in Leibovitz 15 medium (Sigma-Aldrich, St. Louis, MO, USA) supplemented with 2% fetal bovine serum (FBS; Gibco, Waltham, MA, USA), 1% L glutamine (Gibco), 10% tryptose-phosphate (Gibco) and 100 U/ml penicillin, 100 µg/ml streptomycin (Gibco). At 5–7 days post infection, the virus culture supernatants were harvested and concentrated using 40% polyethylene glycol (PEG) 8000 solution (Sigma Aldrich) as described before^39^. The virus concentrate was resuspended in FBS and stored at − 80 °C.

### Foci Reduction neutralization test (FRNT)

Vero cells (ATCC CCL-81) were cultured in Dulbecco’s modified Eagle medium (DMEM; Sigma-Aldrich) supplemented with 5% FBS, and were seeded in 96-well culture plates. Heat-inactivated plasma samples were serially diluted, mixed with equal volume of DENV and incubated for 1 h at 37 °C, 5% CO_2_. Afterwards, plasma-virus mixtures were transferred onto the Vero cells. After 1 h of incubation at 37 °C, the mixture was replaced with 1.8% carboxymethyl cellulose (Sigma-Aldrich) diluted in above-mentioned supplemented DMEM and incubated at 37 °C, 5% CO_2_. After 2–3 days, cells were fixed and stained using mouse anti-DENV polyclonal hyperimmune ascites fluids (IPC). Secondary staining to detect foci was performed using anti-mouse IgG antibody conjugated to horseradish peroxidase (Bio-Rad, München, Germany) and 3, 3′, 5, 5′-tetramethylbenzidine as substrate (Sure- Blue™TMB 1-component microwell peroxidase substrate, medac, Wedel, Germany). The neutralizing antibody titer was expressed as reciprocal of the highest plasma dilution showing ≥ 90% reduction in foci counts (FRNT90 titer) compared to conditions without plasma. FRNT90 curve was generated by non-linear regression analysis using the 4PL sigmoidal dose curve equation on Prism 8 (Graphpad Software). A valid FRNT90 curve required an R^2^ > 0.90, hill slope absolute value ≥ 0.7, and had to reach at least 90% relative infection within the range of the plasma dilutions in the assay. In this assay, 12 paired-plasma samples collected from 12 individuals in cohort 1 and 40 plasma samples in cohort 2 were tested. 1 secondary DF and 2 DHF samples in cohort 2 were excluded after quality control.

### DENV binding IgG assay (DENflow)

A549 cells, a human epithelial carcinoma cell line, were grown in 75 cm^2^ flask of DMEM supplemented with 10% FBS (Gibco), 100 U/ml penicillin, and 100 µg/ml streptomycin (Gibco) and 1% L-glutamine (Gibco) at 37 °C and 5% CO_2_. When the cells reached 70–80% confluency they were inoculated with reference strain DENV-1 or DENV-2 (in 10 ml of RPMI supplemented with 2% FBS) for 90 min at a MOI of 5, depending on the infecting serotype of the patient. Uninfected cells were used as negative control. Afterwards, the cells were washed to remove residual virus and incubated overnight at 37 °C and 5% CO_2_. The cells were harvested and stained with anti-DENV E protein antibody (clone 4G2, ATCC HB-112) labelled with Alexa Fluor 488 (Molecular probes; Thermo Fisher) to confirm infection. For the binding assay, 100 µl of heat-inactivated plasma diluted 1:10 in RPMI were incubated with 50,000 DENV-infected cells/well. The plates were incubated for 30 min on ice. The cells were washed twice with PBS and stained with goat anti-human IgG labeled with Alexa Fluor 647 (Thermo Fisher) to detect IgG antibodies bound to DENV-infected cells. Cells were fixed and analyzed by flow cytometry (FACSCanto II, BD Bioscience). The amount of binding IgG were determined by subtracting percentage of IgG binding to non-infected cells from percentage of IgG binding to DENV-infected cells. Therefore, this assay measures the antibodies that bind to antigens of DENV-1 (cohort 2) or DENV-2 (cohort 1) presented on infected cells. In this assay, 12 paired-plasma samples collected from 12 individuals in cohort 1 and 40 plasma samples in cohort 2 were tested. 1 ASD sample at D10 in cohort 1 was excluded in the final data due to high background staining.

### ADE assay

Human monoclonal antibody G10 (kind gift from Katja Fink, A*STAR, Singapore)^21^, specific for the fusion loop of DENV E protein, and plasma from patients were assessed for ADE activity in human myelomonocyte cell line U937 (ATCC CRL-1593.2). U937 cells were cultured in RPMI (Gibco) supplemented with 10% FBS (Gibco), 100 U/ml penicillin, 100 µg/ml streptomycin (Gibco) and 1% L-glutamine (Gibco). The G10 antibody and heat-inactivated dengue patient plasma was serially diluted fivefold (1:100 to 1:1,562,500) in RPMI with 2% FBS and incubated with each DENV serotype corresponding to MOI of 1 for one hour at 37 °C, 5% CO_2_. Immune complexes were transferred to 96-well round-bottom plates containing 80,000 U937 cells/well. The plates were incubated for 90 min at 37 °C, 5% CO2. Direct infection of U937 cells with DENV in the absence of G10 antibody or plasma was used as control. After infection, cells were washed and incubated for 72 h at 37 °C, 5% CO_2_. Cells were stained with Zombie Aqua viability dye (BioLegend) to distinguish live from dead cells and then fixed, permeabilized and stained for presence of DENV using anti-DENV E protein antibody (clone 4G2, ATCC HB-112) labelled with Alexa Fluor 488 and analyzed by flow cytometry (FACSCanto II, BD Biosience). Percentages of DENV-infected cells were measured and dose-dependent enhancement of infection curve was plotted according percentage of infected cells at each serial dilution. Peak of enhancement titer (PET) was identified as the titer occurring the highest percentage of infection. Area under the curve (AUC) was generated using GraphPad Prism 8 software. In this assay, 18 samples at D0, 15 samples at D10 and 14 samples at D60 collected in cohort 1 and 40 plasma samples in cohort 2 were tested.

### Statistical analysis

Data were analyzed and plotted using GraphPad Prism, version 8.0. The data were tested for normality but did not pass the D’Agostino-Pearson normality test. Therefore, statistical analysis was done using a non-parametric Mann–Whitney test or Friedman test as indicated. Non-parametric Spearman’s method was used for correlation analyses. For all analyses, *p* < 0.05 was considered significant.

## Results

### Patient cohorts

In order to understand the kinetics of the polyclonal antibody response during primary and secondary infection in hospitalized and asymptomatic dengue infected individuals and their relative contribution to protection or severe disease we included 58 DENV*-*infected Cambodian children, in two separate cohorts. Cohort 1 (*n* = 18) consisted of 5 ASD and 13 hospitalized dengue fever (DF) cases and were all classified as secondary infected patients with DENV-2. Cohort 2 (*n* = 40) consisted of both primary and secondary dengue infected individuals, including ASD cases (*n* = 8) and dengue patients classified as classical DF (*n* = 13), DHF (*n* = 13) and DSS (*n* = 6)^34^. Thirty-eight infected-individuals (92.5%) had a confirmed DENV-1 infection while in 4 individuals (7.5%) the infecting serotype could not be determined. The plasma samples were collected at three different time points, at D0, the day of laboratory confirmation of infection and at D10 and D60 after laboratory confirmation. Hospitalized patients were included and sampled after 1–6 days of fever onset (D0). Demographic information and laboratory characteristics from the participants are summarized in Table 1 (cohort 1) and Table 2 (cohort 2).

### Total ADE activity calculated as area under the curve (AUC) is the highest against the infecting serotype during secondary heterotypic infection

**Figure 1:**
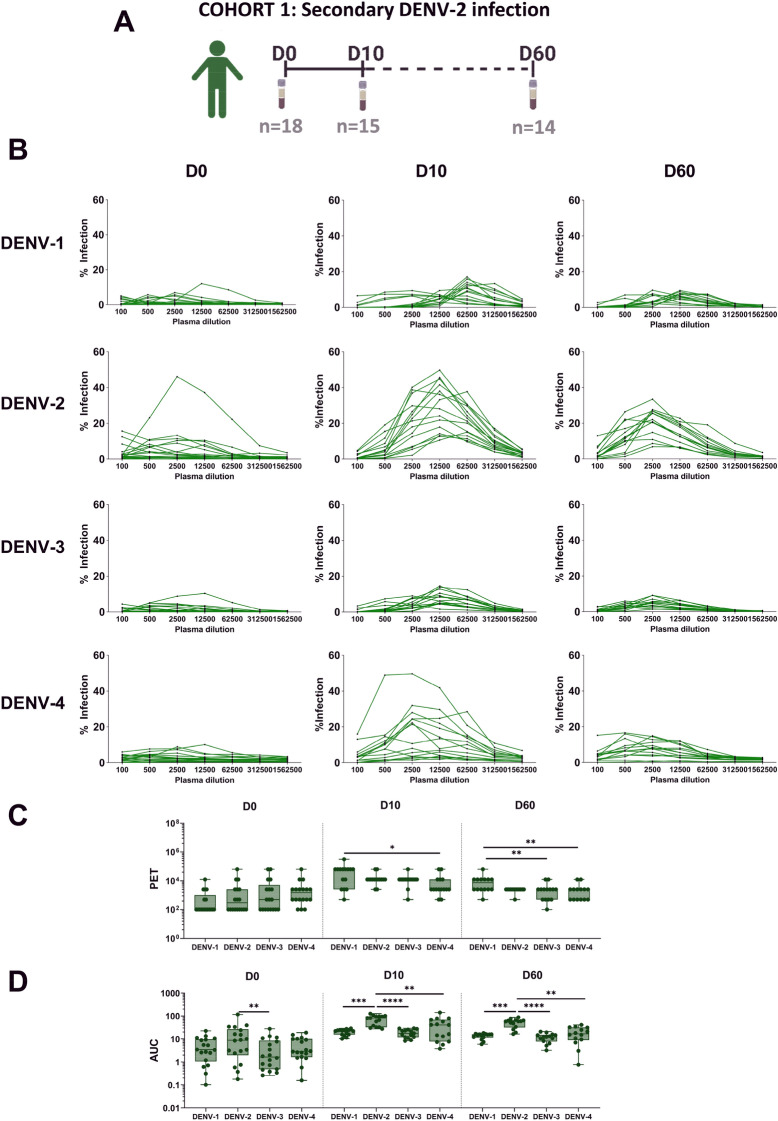
Kinetics of antibody-dependent enhancement (ADE) activity to all four DENV serotypes. Graphical summary of plasma samples collected in cohort 1 (**A**). 18 plasma samples were collected on the day at inclusion (D0, day of laboratory confirmed infection), D10 and D60. There are 12 paired-plasma samples across the 3 time points. Plasma was analyzed in vitro using U937 cells infected with one of the four DENV serotypes (DENV-1 to 4). The percentages of DENV-infected cells in the presence of serial diluted plasma is shown and each line represents an individual plasma sample (**B**). ADE titers are shown as peak of enhancement titer (PET) and area under the curve (AUC) generated from each plasma (**C, D**). Each point in the box plot represents an individual plasma sample. Whiskers show max, min, median values and the interquartile range beyond the 25th and 75th percentiles. Friedman test was used to compare multiple groups in each time point (*P* < 0.05; **P < 0.01; ***P < 0.001; ****P < 0.0001).

We assessed the impact of the polyclonal pool of pre-existing and newly formed anti-DENV IgG to ADE in the acute phase of infection. In order to understand if ADE is mainly directed against the infecting serotype or against other serotypes we performed in vitro ADE assays measuring infection of FcγRI and FcγRII expressing U937 cells^40,41^ in the presence of serially diluted plasma obtained at D0, D10 and D60 after PCR-confirmed DENV-2 infection in cohort 1 (Table 1, Fig. 1A, Figure S1). Each plasma was assessed for ADE activity against four serotypes (DENV-1 to 4). Dose-dependent enhancement of infection curves was obtained against the four DENV serotypes (Fig. 1B). No differences were observed in peak enhancement titer (PET) against four serotypes at the early phase of infection while PET was significantly higher against DENV-1 compared to DENV-4 at late time points, D10 and D60 (Fig. 1C). However, total of ADE activity calculated as area under the curve (AUC) was higher against DENV-2, which is the infecting serotype in the investigated cohort, compared to DENV-1, DENV-3 and DENV-4 at D10 and D60 (Fig. 1D). Overall, these data indicate that plasma from secondary infected patients gradually induced ADE activity against the infecting serotype.

### Antibody titers against the infecting serotype peak at D10 following RT-PCR confirmed infection

**Figure 2:**
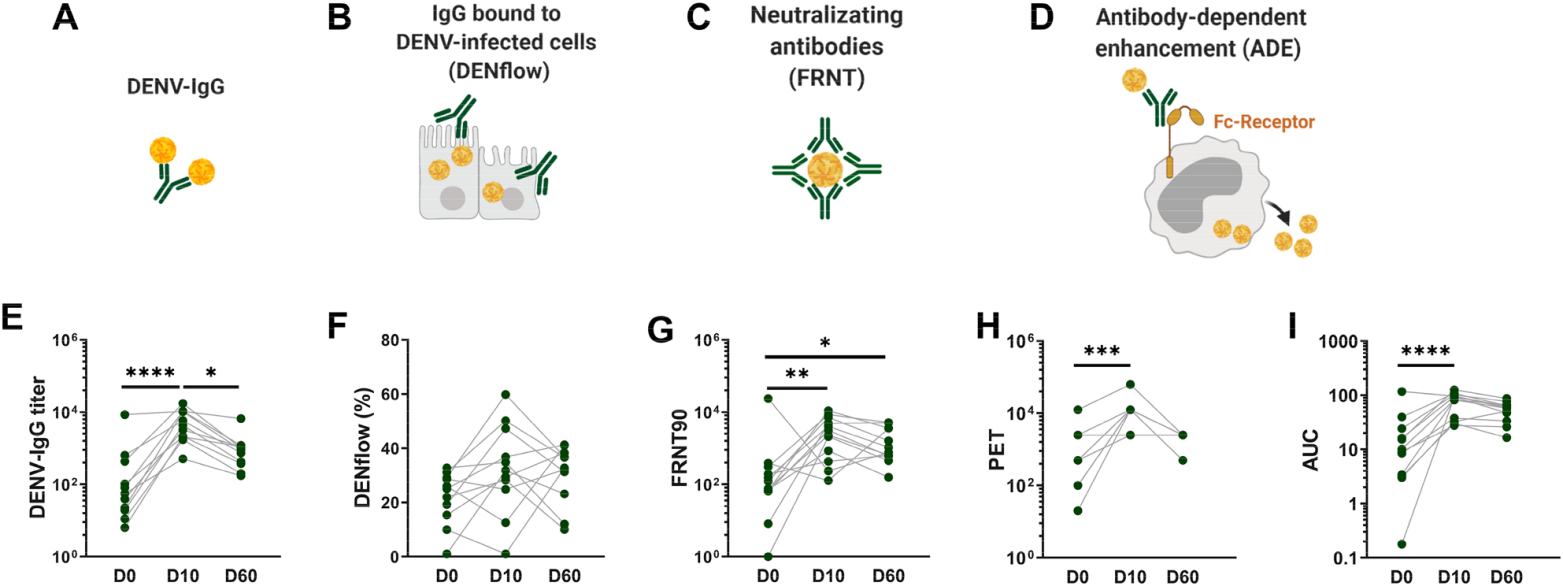
Longitudinal assessment of the anti-DENV antibody responses in paired plasma after secondary DENV-2 infection. Graphical summary of the assays performed in the study including anti-DENV IgG by ELISA assay (**A**), amount of IgG bound to DENV-infected A549 cells by DENflow assay (**B**), neutralizing antibodies by FRNT assay on Vero cells (**C**) and ADE assay on U937 cells (**D**). Paired-plasma samples at different time points (D0, D10 and D60) from twelve individuals infected with DENV-2 in cohort 1 were analyzed for anti-DENV IgG titer (**E**), percentage of IgG binding to DENV-2-infected cells (DENflow) (**F**), neutralizing antibodies (FRNT90) to DENV-2 (**G**) and ADE activity to DENV-2 as PET and AUC (**H, I**). Each point in the graphs represents an individual sample. Friedman test was used to compare three different time points each other (*P < 0.05; **P < 0.01; ***P < 0.001; ****P < 0.0001).

In order to assess the dynamics of the polyclonal antibody response following secondary DENV-2 infection, we measured anti-DENV IgG titers by ELISA, neutralizing antibodies to DENV-2 by FRNT90 and IgG bound to the surface of cells infected with DENV-2 by DENflow assay in patients from cohort 1, next to ADE as described above (Fig. 2A–D). Anti-DENV IgG titers in the plasma peaked at D10 and decreased by D60 (Fig. 2E). The DENflow assay measures the amount of IgG bound to surface of DENV-2 infected cells by flow cytometry, reflecting in vivo conditions of IgG attached to DENV-infected cells (Fig S2). This assay will therefore measure antibodies that are capable of inducing antibody-effector functions such as antibody-dependent cellular cytotoxicity or antibody-depended phagocytosis of infected cells, which are all important mechanisms contributing to clearance of virus-infected cells and innate immune activation^42^.

However, no differences could be observed between the percentages of DENV-infected cells that bound anti-DENV IgG at D0, D10 and D60 post-infection (Fig. 2F). Neutralizing antibodies against DENV-2 as measured by the FRNT90 assay increased from D0 to D10 and remained stable until D60 post-infection. In parallel, ADE titers measured as PET and overall ADE activity as measured as AUC peaked at D10 and remained stable or decreased slightly until D60 post-infection (Fig. 2G–I). Taken together, these results suggest that the quantity and quality of antibody responses to DENV infection peaks at D10 and changes over time in individuals after secondary DENV-2 infection.

### Comparison of anti-DENV antibody responses in infected individuals with different immune status and clinical outcome

**Figure 3:**
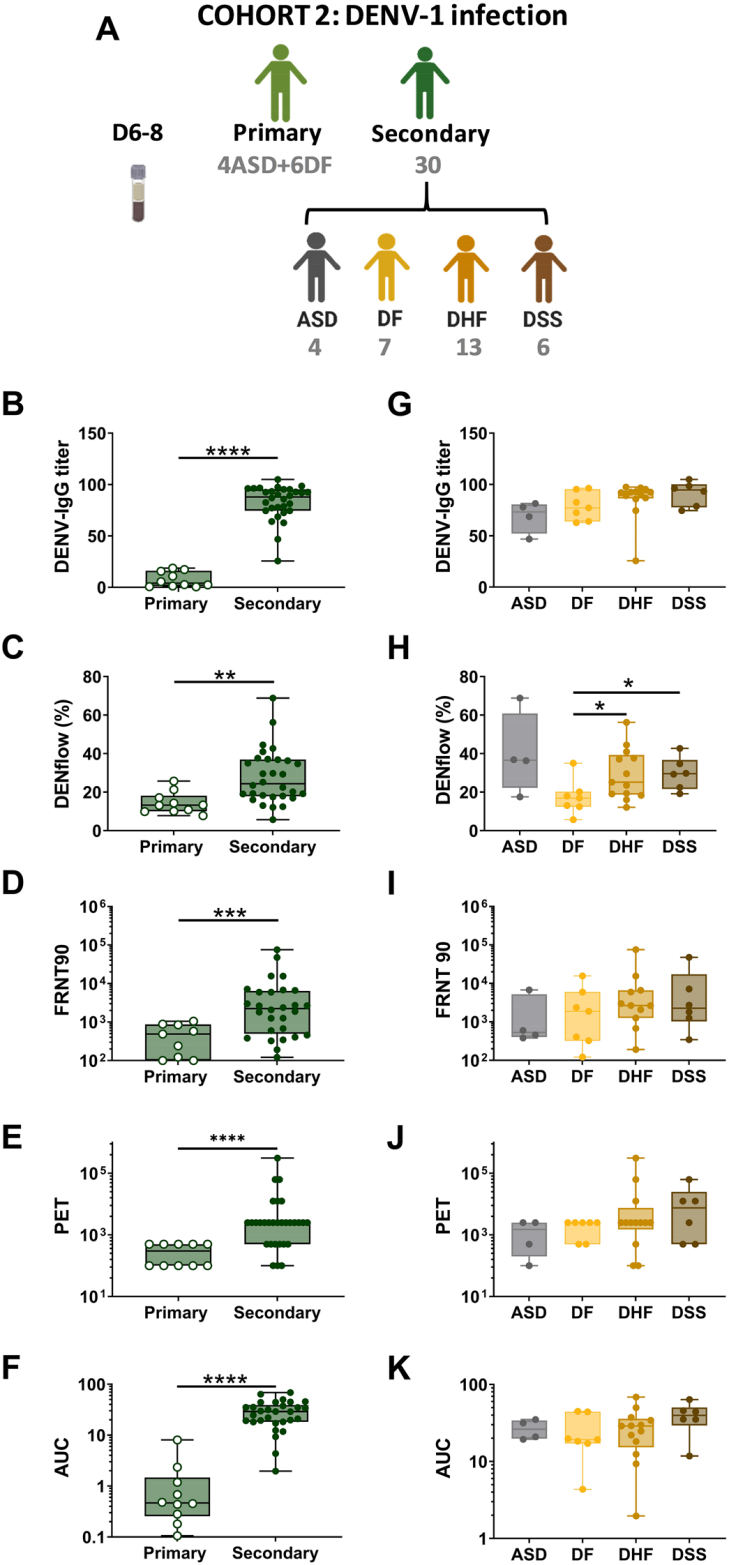
Comparison of anti-DENV antibody responses in infected individuals with different immune status and clinical outcome. Graphical summary of plasma samples collected in cohort 2 (**A**). Plasma samples were collected at day 6 to day 8 after the day after laboratory-confirmed infection. Plasma samples in cohort 2 were analyzed for DENV IgG titer (**B, G**), percentage of IgG binding to DENV-1-infected cells (DENflow) (**C, H**), neutralizing antibodies (FRNT90) to DENV-1 (**D, I**) and ADE activity to DENV-1 expressed as PET (**E, J**) and AUC (**F, K**). Each point in the box plot represents an individual plasma sample. Whiskers show max, min, median values and the interquartile range beyond the 25th and 75th percentiles. Mann–Whitney test was used to compare groups. (*P < 0.05; **P < 0.01; ***P < 0.001; ****P < 0.0001).

As secondary infection results in an increase in anti-DENV IgG titers, we wanted to confirm that these antibodies result in an increase in functional antibodies as well as measured by their neutralizing, binding and enhancing capacities. Hence, we analyzed these features in a second cohort of DENV-1 infected patients close to the peak of antibody titer, between 6 and 8 days after laboratory-confirmed infection, in patients undergoing either primary or secondary DENV infection and in patients with different disease outcome (Fig. 3A). As expected, DENV-IgG titer, FRNT90 titers, IgG bound to infected cells and ADE activity shown as PET and AUC were significantly higher in secondary infected patients compared to primary infected patients. (Fig. 3B–F). Within secondary infected patients, no differences could be observed among ASD, DF, DHF and DSS cases in terms of DENV-IgG titer (Fig. 3G). However, the percentages of DENV-infected cells that bound anti-DENV IgG was decreased in patients with DF compared to patients with DHF, DSS or asymptomatic infected individuals (Fig. 3H). Moreover, both titers of neutralizing antibodies and ADE titers tended to be increased in more severe patients, albeit no significance could be reached (Fig. 3I–K). Taken together, the magnitude of the functional antibody response was associated with immune status, while only the level of IgG bound to DENV-infected cells was associated with disease severity in hospitalized patients between 6 and 8 days after laboratory-confirmed DENV-1 infection.

### The proportion of binding, neutralizing and enhancing antibodies in total anti-DENV IgG is increased in patients with a primary DENV infection

**Figure 4:**
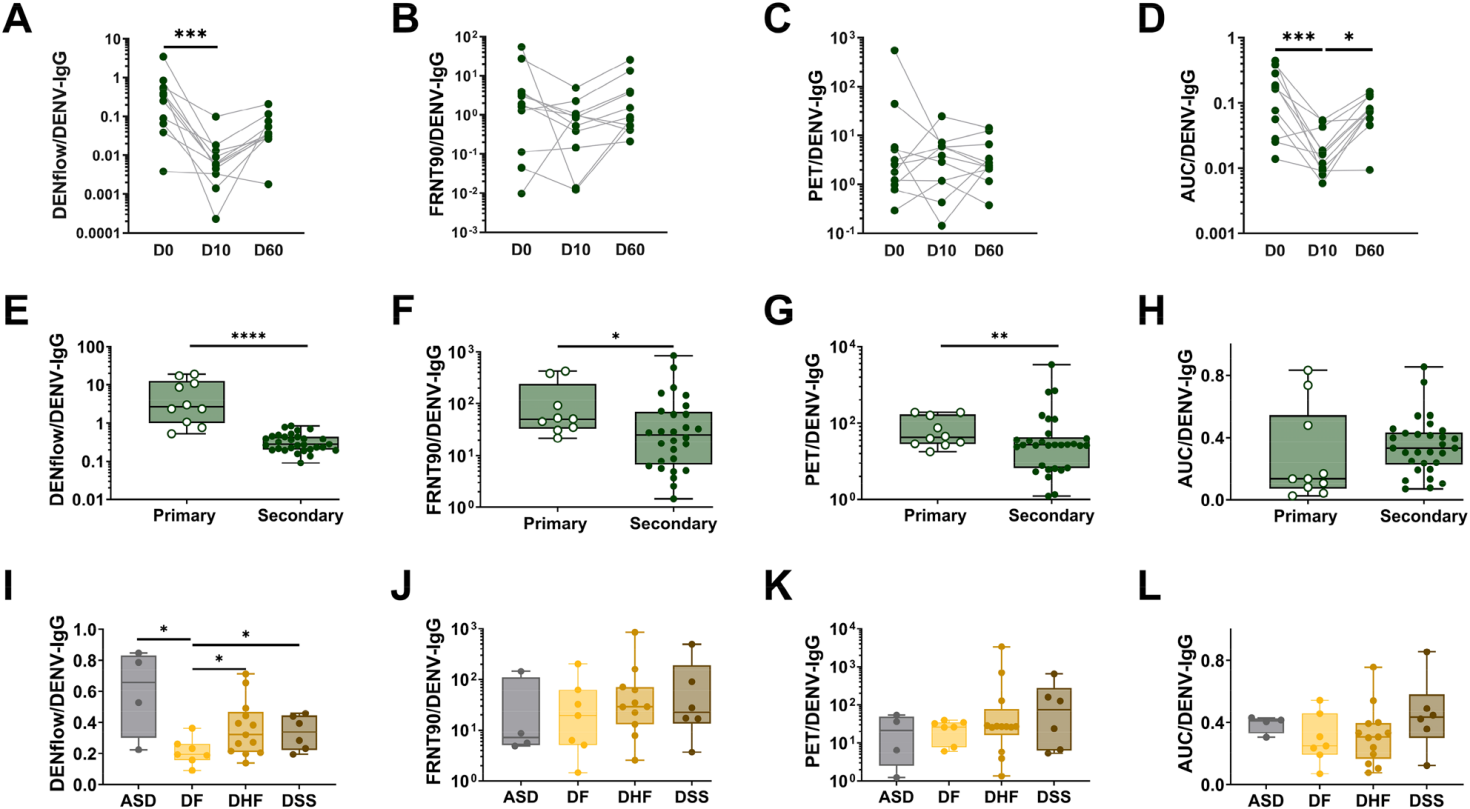
Proportions of neutralizing antibodies and enhancing antibodies within the total DENV-IgG titer. Proportions of DENflow, FRNT90, PET or AUC within the total anti-DENV IgG antibodies in plasma obtained from paired-secondary samples at different time points (D0, D10 and D10) from cohort 1 (**A-D**), in plasma obtained from primary infected patients compared to secondary infected patients from cohort 2 (6–8 days after laboratory-confirmed infection) (**E–H**); and in plasma obtained from secondary infected dengue cases with different disease outcome from cohort 2 (6–8 days after laboratory-confirmed infection) (**I**–**L**). Whiskers show max, min, median values and the interquartile range beyond the 25th and 75th percentiles. Mann–Whitney test was used to compare two patient groups (*P < 0.05; **P < 0.01, ***P < 0.00; ****P < 0.0001).

Neutralization and ADE do not only depend on the quantity of anti-DENV antibodies, but also on the quality of these antibodies. Hence, we assessed if a difference in the proportion of binding, neutralizing or enhancing antibodies was associated with either protection or the development of severe disease after DENV infection. We calculated the ratio of IgG bound to infected cells, neutralizing or enhancing antibodies to total anti-DENV IgG. The amount of IgG bound to infected cells, and AUC (but not PET) titers within the total anti-DENV IgG were higher at D0 compared to D10 and D60 (Fig. 4A,C,D) while the proportions of FRNT90 titers within total DENV-IgG titer were not different among the time points (Fig. 4B). Interestingly, the proportions of IgG bound to infected cells, FRNT90 titers and PET titers within the total anti-DENV IgG were significantly higher in primary infection compared to secondary infection at day 6–9 post laboratory confirmation, (Fig. 4E–H). Within secondary dengue infection, the proportion of IgG bound to infected cells within total anti-DENV IgG was decreased in DF patients compared to other patient groups (Fig. 4I-L). Therefore, these data suggest that the fraction of neutralizing and enhancing antibodies in the total anti-DENV IgG antibodies in primary infected patients contains more neutralizing and enhancing antibodies against the infecting serotype compared to secondary infected patients.

## Discussion

In this study, we aimed to understand the dynamics of the polyclonal antibody responses during the acute phase of secondary infection. Whereas most studies have focused on understanding the role and function of pre-existing anti-DENV antibodies to the outcome of subsequent infection^5,12,43,44^, very few studies have investigated the role of the complex and developing polyclonal anti-DENV IgG antibodies in the outcome of the ongoing infection^45,46^. During acute secondary infection, both pre-existing anti-DENV IgG against the previous infecting serotype and newly formed IgG against either the previous serotype or against the new infecting serotype co-circulate^47,48^. Pre-existing memory B cells raised against the previous infecting serotype will be activated and differentiate to antibody secreting cells (a concept called original antigenic sin)^26,47^. Overall DENV antibody avidity shifts from the previous infecting serotype to the current infecting serotype over time^46^. For all investigated aspects of the humoral response, the functional antibody response has the trend to peak at day 10 after laboratory-confirmed infection, an observation also made in a Nicaraguan cohort^46^.

Serotype-specific neutralization assays remain the gold standard for antibody detection and neutralizing antibodies are used as a proxy for protection, either from infection or at least from clinical disease^6,34^. However, presence of neutralizing antibodies measured after primary infection or vaccination are not always associated with protection during subsequent natural infection in humans. Protective titers can vary according to the serotype, and the quality of the neutralizing response (serotype-specific versus serotype cross-neutralization) is important in defining disease outcome after infection^4,44,49–53^. At 6–8 days after onset of symptoms, which partly coincides with the critical phase of the disease, we do not observe an association between FRNT90 titers measured against the infecting serotype and disease severity in hospitalized patients. No differences were observed in neutralizing antibody titers between secondary infected DF and DHF/DSS patients. However, when comparing neutralizing antibody titers between primary and secondary infected patients we detected higher neutralizing titers after primary infection. Whether this could be one of the factors contributing to a general milder disease outcome after primary infection remains to be established.

We assessed DENV infection enhancing activity of anti-DENV antibodies by an in vitro assay using a FcγRI and FcγRIIa expressing cell line^54–56^. Both PET and AUC were used to evaluate ADE^44,57–59^. While PET provides information of antibody enhancement activity at a particular serum dilution, AUC assesses the magnitude of antibody enhancement activity. In the current study, the range of plasma dilution was appropriate for most plasma samples to observe the peaks of ADE, with a few exceptions coming from some plasma collected at the early time point of the infection. Factors including the choice of FcγR-expressing cell line, virus strains and the ratio between plasma dilution and amounts of virus are important determinants of outcome of the in vitro assay. Neat plasma and diluted plasma were used to observe the kinetics of ADE in previous studies^20,44,60–67^. However, no gold standard ADE assay has been described.

ADE is attributed to either low affinity, serotype cross-reactive antibodies, and/or sub-neutralizing antibody concentrations, or can be attributed to antibodies targeting specific epitopes such as the the prM surface protein or fusion loop of the envelope protein E^17,20–22^. The fraction of neutralizing and enhancing antibodies changes over time due to the evolution of the quality and quantity of anti-DENV antibodies. For example, the amount of afucosylated Fc-IgG increases at convalescence compared to acute infection in primary infection, which might impact antibody dependent enhancement^30,68^ and titers of dengue specific antibody tend to decrease overtime which might favor ADE^18,19^. In our results, ADE is the highest for DENV-2 after secondary DENV-2 infection at day 10, which is also the time point when high neutralization is expected. Indeed, most neutralizing antibodies are able to enhance infection at low concentrations^17–20^.

During an acute DENV infection, a very large population of plasmablasts has been observed, already at time of laboratory diagnosis^48,69,70^. These plasmablasts could be derived either from activated memory B cells and will retain specificy for the primary infection (original antigenic sin) or could be derived from memory B cells that underwent further affinity maturation and selection in a germinal center response and are more cross-reactive or specific for the secondary infection^25,26,47^. Whichever their origin, they could contribute to the increasing concentration of either low affinity or cross-reactive antibodies resulting in increased ADE at D10.

A study in Thailand showed no association of enhancing antibody activity in pre-illness plasma and subsequent disease severity in secondary DENV infection. In a Taiwanese cohort, ADE against the infecting serotype (DENV-2) was assessed during the acute phase and at convalescence. Plasma derived from DHF patients during the acute phase showed higher capacity to induce ADE in vitro than healthy donor plasma^45^. In Cambodia, since 2000 we had alternating outbreaks of DENV-1 (2011–2016), DENV-2 (2002–2004, 2009–2010, 2016–2017) and DENV-3 (2006, 2007)^71^. We report in this study that plasma derived from secondary infected patients compared to primary infection showed higher capacity to induce ADE activity in vitro presented as AUC and PET. In secondary infected patients, we observed that PET titers to the previously circulating serotype (DENV-1) in Cambodia were higher compared to DENV-3 and DENV-4, which had low circulation in Cambodia. Total ADE activity presented as AUC induced by secondary plasma was preferentially directed to the infecting serotype (DENV-2) and increased over time.

Beyond neutralization, antibodies bind to infected cells and exert a range of effector functions, mediated through the antibody Fc part, which aids in the elimination of virus-infected cells. Antibodies bound to infected cells can engage to effector cells expressing Fc-receptors such as monocytes and NK cells. We measured percentages of antibodies bound to DENV-infected cells with an easy to perform binding assay mimicking more closely in vivo antibody binding in patients compared to an ELISA format. The assay measures antibodies that bind to the native confirmation of envelop protein (E) and non-structural protein 1 (NS1) expressed on the surface of infected cells or cells binding NS1^72–77^. These antibodies are important to initiate effector functions, such as antibody-dependent cellular cytotoxicity, antibody dependent complement activation and antibody dependent phagocytosis, which results in antibody-mediated killing of infected cells and activation of innate immune cells^42^. Viral surface proteins such as E and prM can be attached to the surface of infected cells at high concentration, together with NS1^72,74^. Therefore, antibodies with lower avidity to prM and E, as well as anti-NS1 antibodies might be better detected using DENflow compared to an ELISA format. We observed that increased percentage of IgG bound to infected cells 6–8 days after laboratory-confirmed infection is associated with more severe disease outcome in hospitalized dengue patients. These data indicate that binding antibodies might contribute to protection or pathogenesis via other mechanisms than neutralization or antibody-dependent enhancement, such as antibody effector functions and activation of innate immune cells via engagement of FcγR^42^.

One caveat of the study design is that it is impossible to assess the exact timing of infection in asymptomatic dengue cases, which could influence the magnitude of the measured humoral immune response. However, comparable viral loads were detected in asymptomatic and hospitalized dengue cases at inclusion, even though kinetics of viral clearance might be different in asymptomatic cases compared to symptomatic patients^78^. We also acknowledge the small sample size for the patients, which may weaken the power in statistical tests and make the interpretation of these results difficult, especially in the asymptomatic cases. Even with extensive household investigations, it remains a challenge to identify these individuals^31,32^. Therefore, larger sample sizes are needed in further studies to validate the conclusions.

In conclusion, we have assessed the kinetics of the functional antibody response in infected hospitalized patients and asymptomatic infected individuals during the acute and early/late convalescent phase of DENV infection. Anti-DENV IgG titers, neutralization capacity and ADE activity are different between primary and secondary infected hospitalized cases but not between asymptomatic cases and hospitalized patients at the time of laboratory-confirmation of the DENV infection. Titers of neutralizing antibodies and ADE-mediating antibodies against the infecting serotype evolved over time and but were not correlated with disease severity. Total ADE activity induced by secondary plasma was preferentially directed to the infecting serotype (DENV-2) and increased over time. Percentages of IgG bound to DENV-infected cells as measured by DENflow are associated with severity in hospitalized patients. Taken together, these data suggest that binding antibodies might contribute to pathogenesis via other mechanisms than neutralization or enhancement of infection. Measurement of the totality of this response, including the proportion of enhancing antibodies within the total anti-DENV IgG response and determination of IgG binding to DENV-infected cells will give us more insight into humoral immune response to natural infection and vaccine candidates and can identify correlates of protection.

## Data availability

All data are available in the main text or the supplementary materials. All materials, except for clinical specimens, are available on request after completion of a materials transfer agreement with Institut Pasteur du Cambodge.

## Supplementary Information

Supplementary Information.
