# Supplementary material for: Temporal patterns of functional anti-dengue antibodies in dengue infected individuals with different disease outcome or infection history
Source: Sci Rep. 2022 Oct 25;12:17863. doi: 10.1038/s41598-022-21722-2 (PMC9596418; doi:10.1038/s41598-022-21722-2)

**Supporting information**

**
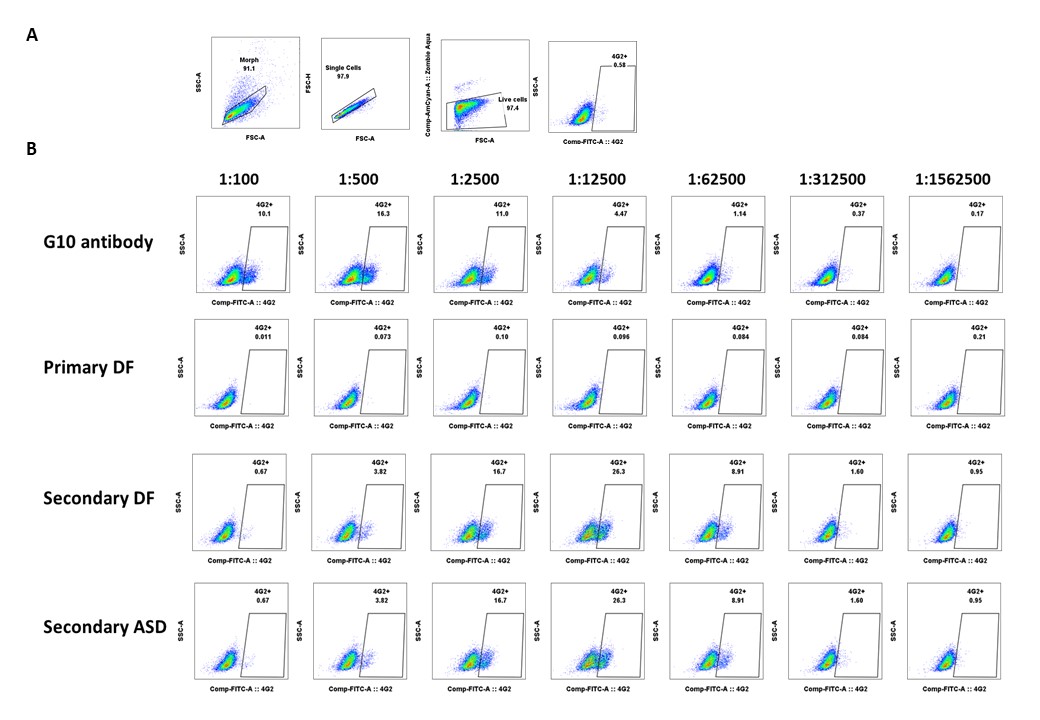
Supplementary Figure S1. Representative flow cytometry plots of *in vitro* ADE.** FcγRI and FcγRII expressing U937 cells were infected *in vitro* with the infecting serotype (DENV-2) in presence or absence of serially diluted plasma or G10 monoclonal anti-DENV Envelop IgG antibody as a positive control. Infected cells were stained using AF488-labeled anti-E protein fusion loop antibody (clone 4G2).

**Supplementary Figure S2. Representative flow cytometry plots of DENflow measuring binding of IgG in cohort 1 to DENV-infected A549 cells.** A549 cells were infected *in vitro* with DENV-2 at MOI-5. After infection the cells were incubated with the plasma. The cells were washed and stained with goat anti-human IgG labeled with Alexa Fluor 647 (Thermo Fisher) to detect IgG antibodies bound to DENV infected cells.


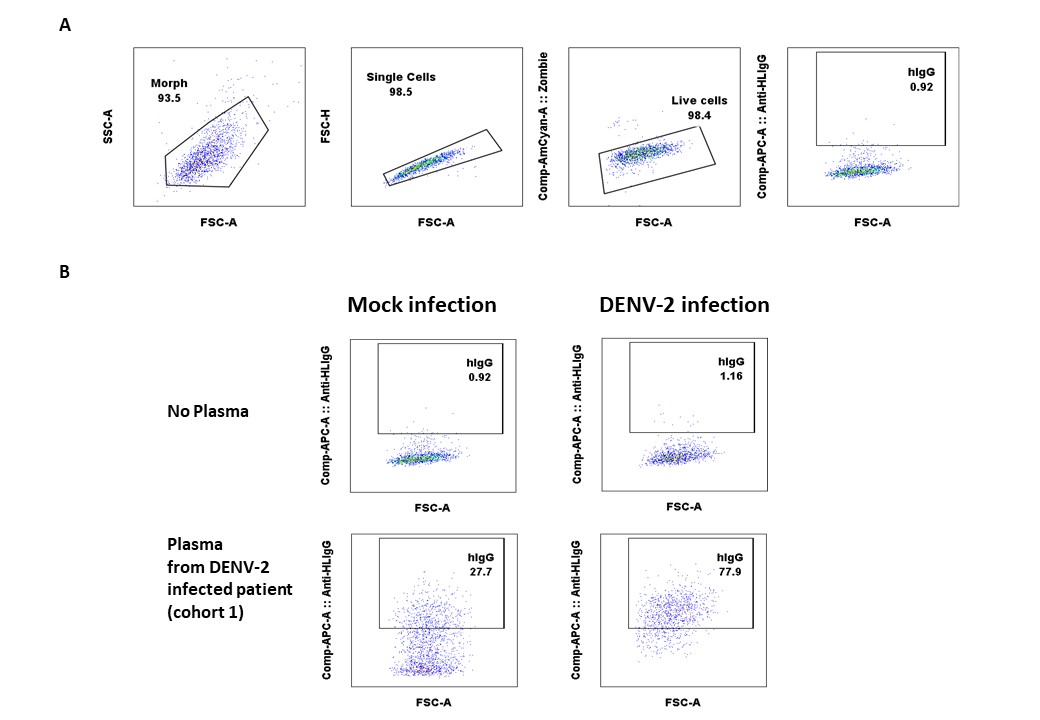

Supplement: Supplementary file 1 — Supplementary Information. [file 41598_2022_21722_MOESM1_ESM.docx]
